# Supplementary material for: Drugs in blood and urine samples from victims of suspected exposure to drink spiking: A prospective observational study from Oslo, Norway
Source: PLoS One. 2024 Jul 10;19(7):e0306191. doi: 10.1371/journal.pone.0306191 (PMC11236145; doi:10.1371/journal.pone.0306191)
Supplement: S1 File — Reporting limits in blood samples and cut-off values in urine samples. (PDF) [file pone.0306191.s002.pdf]

S1 Supplementary table 1. Reporting limits in blood samples

Substance	nmol/L
Alprazolam	10
Amphetamine	200
Benzoylecgonine	200
Buprenorphine	2
Clonazepam	4
Cocaine	50
Codeine	30
Diazepam	200
MDMA	200
Methadone	60
Methamphetamine	200
Morphine	30
Nitrazepam	50
Oxazepam	600
THC	2
Tramadol	200
Zolpidem	70
Zopiclone	20
GHB	100 µmol/L
Ethanol	0.02 ‰

Reporting limits for the project is equal to or higher than published limits of quantitation.
GHB: gammahydroxybutyrate; MDMA: methylenedioxymethamphetamine; THC: tetrahydrocannabinol.

S1 Supplementary table 2. Cut-off values in urine samples

Substance	ng/mL
Amphetamines	300
Benzodiazepines	200
Cocaine	150
Ecstasy	500
Opiates	300
THC	25
GHB	10 µg/mL
Ethanol	0.2 ‰

Ecstasy: methylenedioxymethamphetamine (MDMA) and related substances (methylenedioxyamphetamine (MDA), methylenedioxyethylamphetamine (MDEA), methylbenzodioxolylbutanamine (MBDB), benzodioxolylbutanamine (BDB), paramethoxyamphetamine (PMA), and paramethoxymethamphetamine (PMMA)); GHB: gammahydroxybutyrate; THC: tetrahydrocannabinol.
